# Notes on black elytron species of *Pyrrhalta* Joannis and the description of a new species from China (Coleoptera, Chrysomelidae, Galerucinae)

**DOI:** 10.3897/zookeys.289.4266

**Published:** 2013-04-12

**Authors:** Rui-E Nie, Da-Kang Zhou, Huai-Jun Xue, Xing-Ke Yang

**Affiliations:** 1 Key Laboratory of Zoological Systematics and Evolution,; 2 Institute of Zoology,; 3 Chinese Academy of Sciences, Beijing, 100101, China; 4 Beijing Botanical Garden, Beijing, 100093, China

**Keywords:** Coleoptera, Galerucinae, *Pyrrhalta*, black elytron, new species, key

## Abstract

Thirteen species of *Pyrrhalta* Joannis, 1865 with black elytron are reviewed. A key to species, photographs of aedeagus and habitus are provided. *Pyrrhalta qianana* **sp. n.** is described from Guizhou, China. *Pyrrhalta martensi* Medvedev & Sprecher-Uebersax, 1999 is newly recorded from China (Tibet).

## Introduction

The genus *Pyrrhalta* Joannis, 1865 is a large, worldwide genus distributed in the Holarctic, Oriental and Australian Regions. The genus was firstly proposed as a subgenus of *Galeruca* Geoffroy, 1762 by Joannis (1865), and *Galeruca viburni* Paykull, 1799 was deemed to be fixed as type species by monotypy. The genus presents serious difficulties in several aspects of its classification and nomenclature. Firstly, this genus is very large including 115 species in the world (Xue and Yang 2010). Secondly, its status is still not entirely clear being obscured by apparent relationship of *Pyrrhalta* and *Galerucella* Crotch, 1873 (Weise 1924, Gressitt and Kimoto 1963, Wilcox 1965). Separation of species of typical *Pyrrhalta* and *Galerucella* was based on a character of the pronotum. The whole pronotum of *Pyrrhalta* is covered by hairs, while at least the middle region of the pronotum of *Galerucella* is glabrous (Gressitt and Kimoto 1963). However, a use of this character was not consistent in the past by different specialists. Some species whose pronotum covered with hair were treated as *Galerucella*, while some species having glabrous area of pronotum were placed in *Pyrrhalta*. Meanwhile, some tentative *Galerucella* species were included into *Pyrrhalta* clade based on molecular data (Nie et al. 2012). Whether the pronotum character can be regarded as the unique character to distinguish the two genera need be further studied, and the morphological characters for distinguishing *Galerucella* and *Pyrrhalta* need to be addressed in the future. Thirdly, the subgenera of *Pyrrhalta* are still not well defined. For example, *Clitenososia* Laboissière, 1931, *Xanthogaleruca* Laboissière, 1934 and *Tricholochmaea* Laboissière, 1932 were considered as synonyms of *Pyrrhalta* (Gressitt and Kimoto 1963), while in another study, the groups *Galerucella*, *Neogalerucella* Chûjô, 1962, *Xanthogaleruca* and *Tricholochmaea* were treated as subgenera of *Pyrrhalta* (Wilcox 1965). *Galerucella*, *Xanthogaleruca*, and *Tricholochmaea* were treated as valid genera and *Neogalerucella* as subgenus of *Galerucella* in some recent studies (Silfverberg 1974, Beenen 2008, Gök et al. 2007, Beenen 2010). The most important argument is whether *Xanthogaleruca* is treated as a valid genus or a subgenus of *Pyrrhalta*. The character that defines *Xanthogaleruca* as a genus is the aedeagus with a comb-shaped internal sac (Silfverberg 1974, Beenen 2003, Gök et al. 2007). However, the relative value of this character is not proved yet. In present study, *Xanthogaleruca* is treated as a synonym of *Pyrrhalta* until evidence suggesting otherwise are presented.

This study focuses on the *Pyrrhalta* species with black elytra, which may not be a natural group. We defined the main character state of the group as “elytra entirely or at least 2/3 black”. There are 13 species included in this group. Among them, *Pyrrhalta qianana* sp. n., a new species described below. A key to the species of the group is provided.

## Material and methods

Morphological characters were examined with an Olympus SZ 61 microscope. Genitalia of males and / or females of each species were dissected using the following procedure: for dried or ethanol preserved specimens, the abdomen was separated, transferred to a vial containing 10% KOH which was heated in a boiling water bath for 10 min. The genitalia were then carefully removed in a cavity slide under distilled water using fine forceps and hooked minuten-pin dissecting needles. Series of partially focused photographs were made with a digital camera (Nikon D300S) attached to a stereomicroscope (Zeiss Discovery V12), and then combined using Helicon Focus software, and finally were evaluated and assembled using Adobe Photoshop CS 8.0 and Illustrator CS4 software.

### Material examined is deposited in the following collections

**BMNH** The Natrual History Museum, London, UK

**IZAS **Institute of Zoology, Chinese Academy of Sciences, Beijing, China

**JBCB **Jan Bezděk collection, Brno, Czech Republic

**MHBU** Museum of Hebei University, Baoding, Hebei, China

**NHMB **Naturhistorisches Museum Basel, Switzerland

**USNM **The United States National Museum of Natural History (Smithsonian Institution), Washington, D.C., USA

**ZIN** Zoological Institute, Russian Academy of Sciences, Saint-Petersburg, Russia

## Taxonomy

### Key to species of *Pyrrhalta* with entirely or at least 2/3 black elytra

**Table:** 

1	Head and pronotum yellowish brown, with or without black markings	2
–	Head and pronotum dark, with or without brown markings	10
2	Occiput without black spots	3
–	Occiput with black spots	4
3	Elytron entirely black with flattish surface, space between punctures smaller than diameter of puncture	*Pyrrhalta huangshana* Chen, 1964
–	Elytron black, suture and lateral margins light yellow; elytral surface convex, space between punctures larger than diameter of puncture	*Pyrrhalta qianana* sp. n.
4	Middle of pronotum with black reversed trapezoid marking	*Pyrrhalta meghalayana* Medvedev, 2002
–	Pronotum with three black spots, located in both lateral sides and middle of disc	5
5	Elytron black, a short yellowish brown longitudinal stripe present at base of elytron, which less than 1/4 of elytral length	*Pyrrhalta martensi* Medvedev & Sprecher, 1999
–	Elytron black, without yellowish brown stripe or with a long yellowish brown longitudinal stripe through the whole elytron	6
6	Elytron surface with ridges	7
–	Elytron surface without ridges	8
7	Elytron dark metallic bronze with slight shade of olive green; with two distinct longitudinal ridges close to suture and flat margin	*Pyrrhalta subaenea* (Ogloblin, 1936)
–	Elytron black, lateral margin and epipleuron brown, disc with 7 irregular black spots formed by groups of dense punctures, short yellowish band present at base of elytron which is the same length as scutellum; longitudinal ridge in middle of disc and under humerus	*Pyrrhalta tianmuensis* Chen, 1964
8	Elytron black, each with yellow longitudinal stripe starting side of humerus and ending before elytral apex, elytral margin and epipleuron brown	*Pyrrhalta warchalowskii* Bezděk, 2007
–	Elytron black or reddish brown or brown black, without yellow longitudinal stripe, elytral margin and epipleuron brown or black	9
9	Antenna 1/3 as long as elytra; basal margin of pronotum slightly concave in middle, lateral margin clearly convex in 1/2; elytron entirely black, disc convex with dense, deep punctures	*Pyrrhalta orientalis* (Ogloblin, 1936)
–	Antenna 1/2 as long as elytra; basal margin of pronotum sinuate, lateral margin narrow basally, obtusely rounded anteriorly; elytron black, basally, along suture, with stripe in middle of disc, lateral margin and epipleuron brown, disc with dense, small, and shallow punctures	*Pyrrhalta sulcatipennis* (Chen, 1942)
10	Occiput with two brown spots; pronotal disc with six small depressions	*Pyrrhalta fossata* (Chen, 1942)
–	Occiput without two brown spots; pronotal disc with three depressions	11
11	Elytron reddish brown with broad longitudinal blackish marking covering nearly all interior 2/3 of disc	*Pyrrhalta tatesuji* Kimoto, 2001
–	Elytron without blackish marking	12
12	Antenna slender; pronotum with V shaped depressions besides of disc, a triangle concave near middle of basal margin; elytron bronzy black with purplish or bronzy tinges metallic lustre, lateral margin not with expanded margin	*Pyrrhalta metallica* Gressitt & Kimoto, 1963
–	Antenna very stout; pronotum with a wide longitudinal depression in the middle of disc, round depressions besides of disc; elytron brown black, different shades, with bronze metallic lustre, lateral margin expanded with a swollen ridge parallel to margin	*Pyrrhalta xizangana* Chen & Jiang, 1981

#### 1. Pyrrhalta fossata

(Chen, 1942)

http://species-id.net/wiki/Pyrrhalta_fossata

Figs 1–2 , 42

Galerucella fossata Chen, 1942: 19.
Pyrrhalta fossata : Gressitt & Kimoto, 1963: 439, 449.
Neogalerucella fossata : Beenen, 2010: 449.

##### Specimens examined.

Type material: Holotype: ♀, re-written (original label is in Chinese): China, Sichuan, Kangding, 25-VIII-1939, collector unknown (IZAS).

##### Distribution.

China (Sichuan).

##### Notes.

Holotype of this species was diagnosed as male in the original description without being dissected (Chen 1942). Actually the holotype is female. Its spermatheca is illustrated here (Fig. 42). The updated catalogue of Galerucinae (Beenen 2010) showed that this species belonged to a subgenus *Neogalerucella* of *Galerucella*. In this study, we found that the disc of pronotum is entirely covered by hairs. So we think that this species should be remained in *Pyrrhalta*.

**Figures 1–12.**
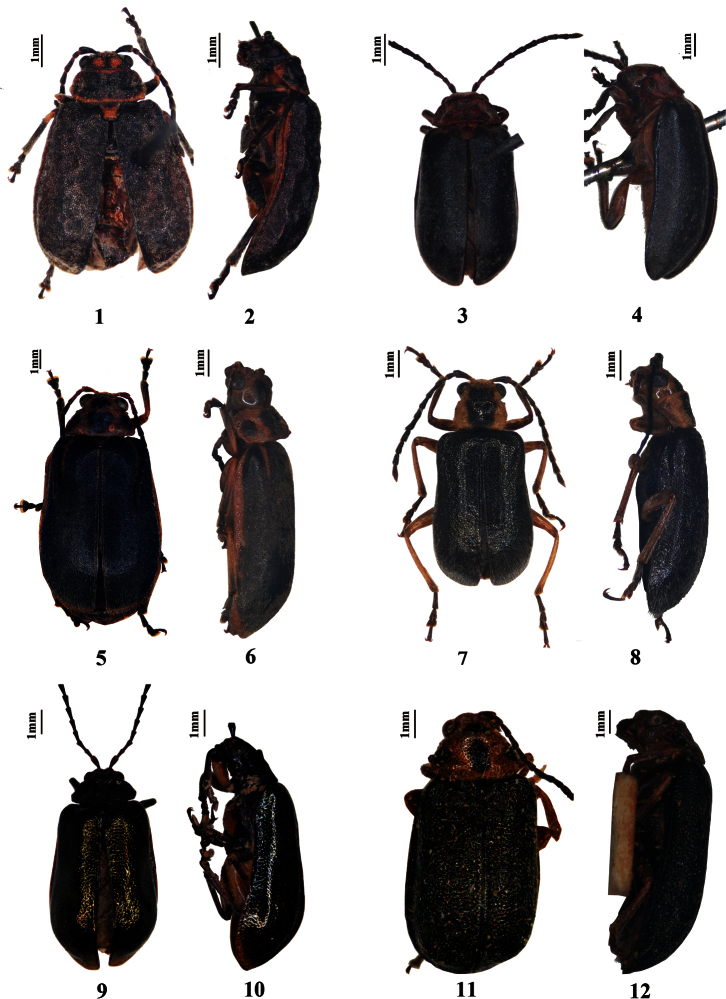
Habitus. **1–2** *Pyrrhalta fossata* (holotype) **3–4** *Pyrrhalta huangshana* (holotype) **5–6** *Pyrrhalta martensi* (paratype) **7–8** *Pyrrhalta meghalayana* (paratype) **9–10** *Pyrrhalta metallica* **11–12** *Pyrrhalta orientalis* (holotype).

#### 2. Pyrrhalta huangshana

Chen, 1964

http://species-id.net/wiki/Pyrrhalta_huangshana

Figs 3–4 , 24–25

Pyrrhalta huangshana Chen, 1964: 207.

##### Specimens examined.

Type material: Holotype: ♂, re-written (original label is in Chinese): China, Anhui, Huang Mountain, 5-VIII-1936, collector unknown (IZAS).

##### Distribution.

China (Anhui).

##### Notes.

Aedeagus of the holotype is illustrated here (Figs 24-25), dorsal view: asymmetrical but nearly parallel-sided, tapered at extreme apex which is subacute; dorsal opening largely on the left side; lateral view: gradually tapering to a subacute tip, slightly arched on the left side, nearly semi - circular on right side.

#### 3. Pyrrhalta martensi

Medvedev & Sprecher, 1999

http://species-id.net/wiki/Pyrrhalta_martensi

Figs 5–6 , 26–27

Pyrrhalta martensi Medvedev & Sprecher, 1999: 366.

##### Specimens examined.

Type material: Paratypes: 1 ♀, original label: “W-Nepal, Kali Gandaki, Knola C.J. Rai / Kopchepani, 1500–1600 m, 21-V-1984 / PARATYPUS, L.N. Medvedev” (NHMB); 1♂, original label: “India, Darjeeling D., Bhakta B. / Kalimpong, 9th mile 1500 m, 14.VII84 / PARATYPUS, L.N. Medvedev”(NHMB).

Other material (2 spec.): 2♂, China, Tibet, Linzhi, Muotuo, Yarang, 760m, 19-VIII-2006, Ming Bai leg. (IZAS).

##### Distribution.

China (Tibet), India, and Nepal.

##### Notes.

This is the first record of this species in China.

#### 4. Pyrrhalta meghalayana

Medvedev, 2002

http://species-id.net/wiki/Pyrrhalta_meghalayana

Figs 7–8 , 28–29

Pyrrhalta meghalayana Medvedev, 2002: 247.

##### Specimens examined.

Type material: Paratypes: 1♂, 1 ♀, original label: “NE INDIA; Meghalayana; 1999, 3Km E Tura; 1150; 25°30'N, 90°14'E; 18. IV.; Dembický & Pacholátko leg. / PARATYPE” (NHMB).

Other material (1 spec.): 1 ♀, NE India, Meghalaya, SW of Sohra, 25°13–14'N, 91°40', 700–950m, 22-V-2005, C.L. Peša leg. (JBCB).

##### Distribution.

India.

#### 5. Pyrrhalta metallica

Gressitt & Kimoto, 1963

http://species-id.net/wiki/Pyrrhalta_metallica

Figs 9–10 , 30–31

Pyrrhalta metallica Gressitt & Kimoto, 1963: 457.

##### Specimens examined.

Type material: Allotype: 1♀, original label: “near O-Er., Nr Weichow, Aug. 6 18 ‘33, 6000–1500 ft / SzechwanChina, DCGraham / ALLOTYPE J.L. Gressitt” (USNMNH).

Other material (3 spec.): 2♂, China, Yunnan,Yundi, 3700m, 28-VII-1979, Jiang-Xing Rao leg. (IZAS); 1♂, China, Yunnan, Yundi, 3700m, 28-VII-1979, Zhi-Wen Kui leg. (IZAS).

##### Distribution.

China (Sichuan, Yunnan).

#### 6. Pyrrhalta orientalis

(Ogloblin, 1936)

http://species-id.net/wiki/Pyrrhalta_orientalis

Figs 11–12 , 32–33

Galerucella (Xanthogaleruca) orientalis Ogloblin, 1936: 102, 390.
Pyrrhalta orientalis : Gressitt & Kimoto, 1963: 461.

##### Specimens examined.

Type material: Type: ♂, original label: “Shan-hai-Kwan, In Mountains, 1.9.06., F.M. Thomson, 1907-200 / *Galerucella orientalis* sp. n., D. Ogloblin det., 1935. type.” (BMNH).

Other material (44 spec.): 1♂, China, Beijing, Badaling, 700m, 23-VII-1964, Qin Zhou (IZAS); 1♀, China, Beijing, Badaling, 27-V-1980, collector unknown (IZAS); 2♀, China, Beijing, Shanbao, 26-V-1978, Sheng-Qiao Jiang leg. (IZAS); 1♂, China, Beijing, Shanbao, 4-VII-1978, Sheng-Qiao Jiang leg. (IZAS); 1♂, China, Beijing, Shanbao, 09-VII-1964, Su-Bo Liao leg. (IZAS); 19♀, 19♂, China, Shandong, Tai Mountain, 21-IV-1993, Cheng-Gang Zhou leg. (IZAS)

##### Distribution.

China (Liaoning, Hebei, Shanxi, Shandong, Fujian).

##### Notes.

Aedeagus is illustrated here (Figs 32–33), dorsal view: slightly asymmetrical, gradually and slightly widened to apex, apex with a long acute tip; lateral view: nearly straight on left side, gradually tapering to acute tip on right side.

#### 7. Pyrrhalta subaenea

(Ogloblin, 1936)

http://species-id.net/wiki/Pyrrhalta_subaenea

Figs 13–14 , 34–35

Galerucella (Xanthogaleruca) subaenea Ogloblin, 1936: 102, 389.
Pyrrhalta subaenea : Gressitt & Kimoto, 1963: 466.

##### Specimens examined.

Type material: Syntypes: 1♀, 1♂, original label: “entre Za-mi et Ta-pa, 16-VII-93, Tchouen tshin / Syntypus” (ZIN).

##### Distribution.

China (Sichuan).

##### Notes.

Aedeagus of a syntype is illustrated here (Figs 34-35), dorsal view: slightly asymmetrical, gradually and slightly widened to apex, apex with acute tip in middle; lateral view: gradually tapering to subacute tip, slightly arched on left side, nearly semi-ellipse on right side.

**Figures 13–23.**
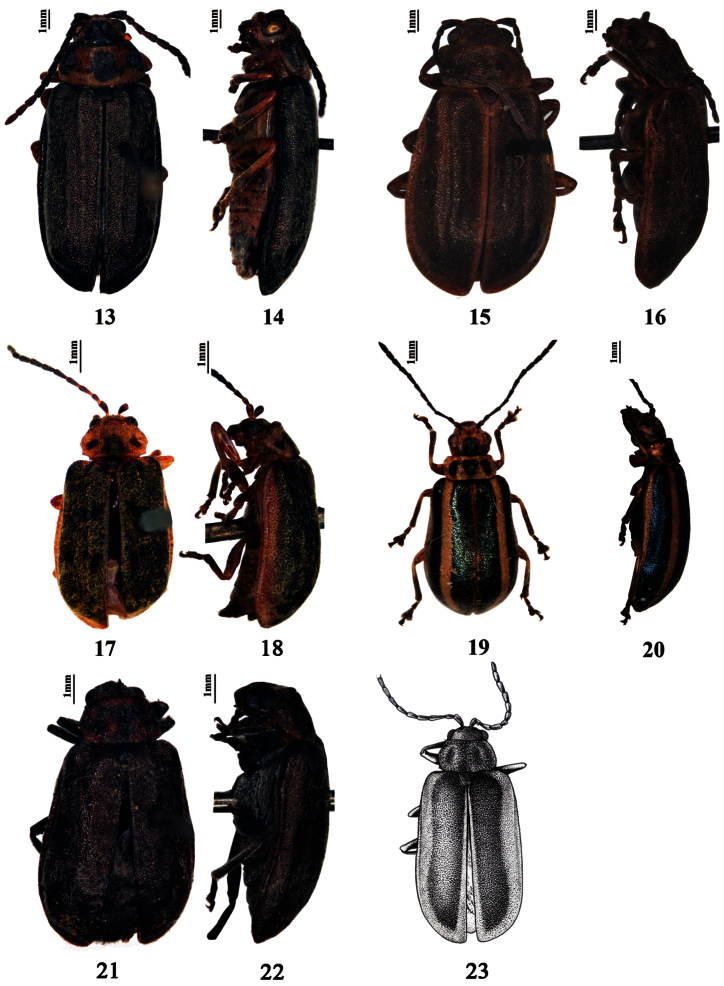
Habitus. **13–14** *Pyrrhalta subaenea* (syntype) **15–16** *Pyrrhalta sulcatipennis* (holotype) **17–18** *Pyrrhalta tianmuensis* (holotype) **19–20** *Pyrrhalta warchalowskii* (paratype) **21–22** *Pyrrhalta xizangana* (holotype) **23** *Pyrrhalta tatesuji* Kimoto (drawing after original photograph in Kimoto 2001).

**Figures 24–35.**
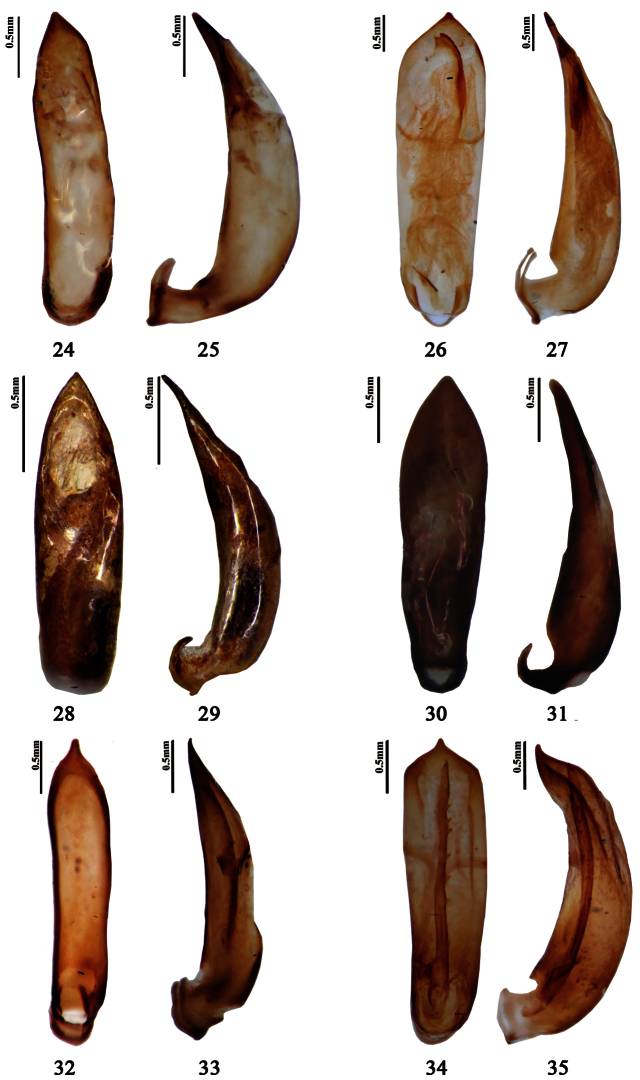
Aedeagus. **24–25** *Pyrrhalta huangshana* (holotype, **24** dorsal view **25** lateral view) **26–27** *Pyrrhalta martensi* (**26** dorsal view **27** lateral view) **28–29** *Pyrrhalta meghalayana* (paratype, **28** dorsal view **29** lateral view) **30–31** *Pyrrhalta metallica* (**30** dorsal view **31** lateral view) **32–33** *Pyrrhalta orientalis* (**32** dorsal view **33** lateral view) **34–35** *Pyrrhalta subaenea* (syntype, **34** dorsal view **35** lateral view).

#### 8. Pyrrhalta sulcatipennis

(Chen, 1942)

http://species-id.net/wiki/Pyrrhalta_sulcatipennis

Figs 15–16 , 36–37

Gallerucella sulcatipennis Chen, 1942: 18.
Pyrrhalta sulcatipennis : Gressitt & Kimoto, 1963: 466.

##### Specimens examined.

Type material: Holotype:♂, re-written (original labels are in Chinese): China, Sichuan, Emei Mountain, 25-VIII-1939, C.S. TSI leg. (IZAS); Paratype: 1♀, 1♂, the same locality as Holotype (IZAS).

Other material (26 spec.): 1♀, China, Hunan, Sangzhi, Tianping Mountain, 1400m, 12-VIII-1988, Shu-Yong Wang leg. (MHBU); 5♀, 5♂, China, Hunan, Sangzhi, Bada Mountain, 10/11-VII-2004, Ji-Liang Wang and Jian-Feng Wang leg. (MHBU); 4♂, China, Sichuan, Emei Mountain, 25-VIII-1939, You-Cai Lu and Zong-Yuan Wang leg. (IZAS); 2♀, China, Guizhou, Fanjing Mountain, 2300m, 5/4-VIII-2001, Hong-Bin Liang leg. (IZAS); 6♀, China, Guizhou, Fanjing Mountain, 800–1600m, 5-VIII-2001, Kang-Zhen Dong leg. (IZAS); 3♂, China, Guizhou, Zunyi, Kuankuoshui Nature Reserve, 1530m, 7-VI-2010, Rui-E Nie leg. (IZAS).

##### Distribution.

China (Hunan, Sichuan, Guizhou).

**Figures 36–43.**
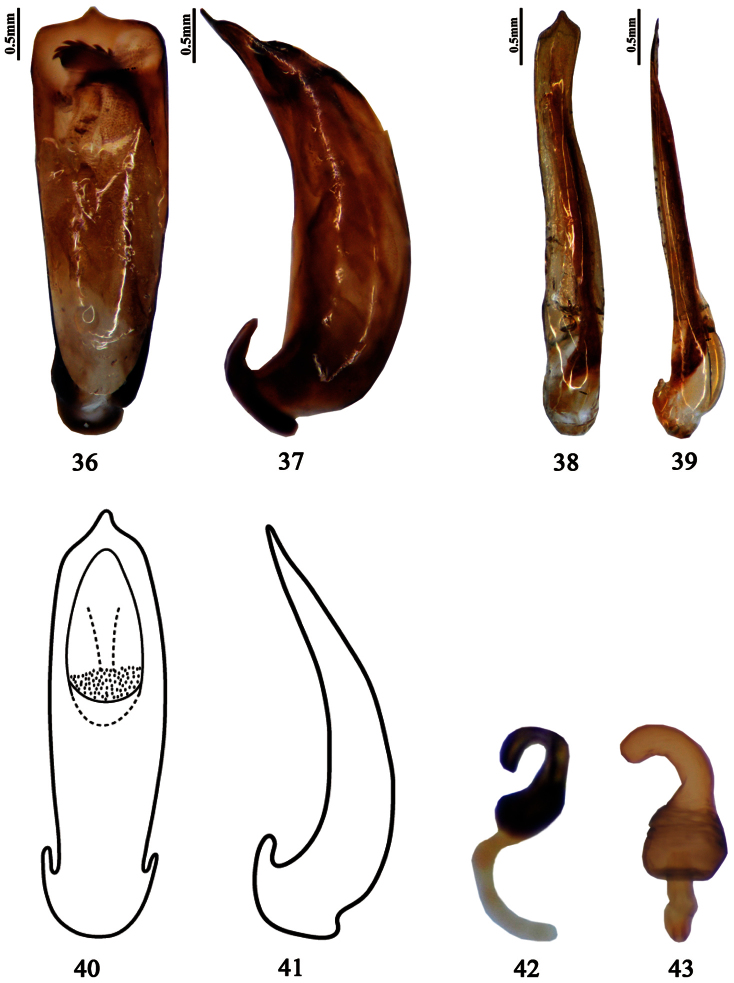
Aedeagus / spermatheca **36–37** *Pyrrhalta sulcatipennis* (holotype, **36** dorsal view **37** lateral view) **38–39** *Pyrrhalta tianmuensis* (**38** dorsal view **39** lateral view) **40–41** *Pyrrhalta warchalowskii* (Bezděk 2007 orig., **40** dorsal view **41** lateral view) **42** spermatheca of *Pyrrhalta fossata* (holotype) **43** spermatheca of *Pyrrhalta xizangana* (paratype).

#### 9. Pyrrhalta tatesuji

Kimoto, 2001

http://species-id.net/wiki/Pyrrhalta_tatesuji

Fig. 23

Pyrrhalta tatesuji Kimoto, 2001: 45.

##### Distribution.

Nepal.

##### Notes.

We did not examine any specimens of this species. We diagnosed it as belonging to elytron-black group based on the original description and the figure of type (Fig. 23).

#### 10. Pyrrhalta tianmuensis

Chen, 1964

http://species-id.net/wiki/Pyrrhalta_tianmuensis

Figs 17–18 , 38–39

Pyrrhalta tianmuensis Chen, 1964: 207.

##### Specimens examined.

Type material: Holotype: ♀, re-written (original labels are in Chinese): China, Zhejiang, Tianmu Mountain, 6-VIII-1937, collector unknown (IZAS).

Other material (1 spec.): 1♂, China, Zhejiang, W. Tianmu Mountain, 30-VII-1998, Hong Wu leg. (IZAS).

##### Distribution.

China (Zhejiang).

##### Notes.

Aedeagus is illustrated here for the first time. Aedeagus: slender, dorsal view: strongly asymmetrical, apex with acute tip, apical part narrower than basal part, gradually tapering apically but arching near half of base on right side, nearly straight on left side before apex; lateral view: very slender, nearly straight on left side, suddenly tapering to acute tip at basal 1/4 on right side (Figs 38–39).

#### 11. Pyrrhalta warchalowskii

Bezděk, 2007

http://species-id.net/wiki/Pyrrhalta_warchalowskii

Figs 19–20 , 40–41

Pyrrhalta warchalowskii Bezděk, 2007: 607.

##### Specimens examined.

Type material: Paratype: 1♀, original label: “S-INDIA, Tamil Nadu state, Nilgiri hills, 10km SW of Manjoor, 76°35'E, 11°12'N, Thiashola reserved forest / near Carrington estate, ca 2100m, 14-19-VI-1999, Z. Kejval & M. Trýzna leg. / PARATYPUS” (JBCB).

##### Distribution.

India.

#### 12. Pyrrhalta xizangana

Chen & Jiang, 1981

http://species-id.net/wiki/Pyrrhalta_xizangana

Figs 21–22 , 43

Pyrrhalta xizangana Chen & Jiang, 1981: 459.

##### Specimens examined.

Type material: Holotype:♂, re-written (original labels are in Chinese): China, Tibet, 52 Daoban, 9-VII-1976, Yin-Heng Han leg. (IZAS). Paratypes: 3♀, re-written (original labels are in Chinese): China, Tibet, Chaya, Jitang, 7-VII-1976, Yin-Heng Han leg. (IZAS).

##### Distribution.

China (Tibet).

#### 13. Pyrrhalta qianana

Nie & Yang sp. n.

urn:lsid:zoobank.org:act:0ABCCB95-FF41-4DE8-9185-E2B29454998C

http://species-id.net/wiki/Pyrrhalta_qianana

Figs 44–49

##### Type material.

Holotype: ♂, China, Guizhou, Zunyi, Kuankuoshui Nature Reserve, Baishaogou, 9-VI-2010, Wan-Gang Liu leg. (IZAS). Paratype: 1♂, the same data as holotype (IZAS); 1♂, China, Sichuan, Fengdu, Shiping, 610m, 3-VI-1994, Wen-Zhu Li leg. (IZAS); 1♂, China, Chongqing, Beipei, Tuanjie, 6-V-1999, Hai-Jian Wang and Yin-Fei Zhu leg. (IZAS); 1♀, China, Guizhou, Jiangkou, Huixiangping, 2-III-2001, Guo-Dong Ren leg. (MHBU); 1♀, China, Sichuan, Emei Mountain, 1800-1900m, 14-VIII-1957, Fu-Xing Zhu leg (IZAS).

##### Diagnosis.

Thisspecies can be separated from all known species in the genus by the following characters: very long antennae (length=4.9 mm), antennomere 3 more than 2 times as long as antennomere 2, and last abdominal sternite of male with very deep U-shape cavity (Fig. 46).

##### Description.

Generally black, apex of labrum and mandible, maxilla, dark brown; head, pronotum, elytral margin, elytral suture, yellowish; antenna black except ventral side of antennomeres 1-5; legs brown except ventral sides of tibiae and tarsi black; scutellum yellowish brown, dark brown on basal part. Body densely covered with short pale silvery pubescences.

Head slightly narrower than prothorax; occiput flat; epicranial suture distinct; frontal tubercles distinctly raised, subquadrate, vertex impunctate.

Antennae long, slender, 0.85× as long as body, length ratio of antennomeres 1 to 11: 15-11-24-23-22-20-20-16-19-17-20.

Pronotum transverse, nearly 2× as broad as long, maximum width across pronotum 1.55 mm, distance from basal margin to anterior margin 0.75 mm, anterior margin nearly straight and slightly emarginate at middle, lateral margin constricted in anterior third, basal margin slightly concave mesally; anterior angle nearly rectangular, and posterior angles obtusely rounded. Surface densely pubescent, irregularly punctured, with pair of deep depressions laterally and longitudinal depression in middle.

Scutellum trapezoid, densely punctured, sparsely pubescent.

Elytron subparallel, nearly 3.7× as long as broad, maximum width across both elytra 1.15 mm, linear distance from base to apex of elytra 4.25 mm; surface confusedly punctured and closely covered with fine hairs; space between punctures smaller than diameter of puncture; epipleuron slightly broad basaly, gradually narrowed toward apex.

Ventral surface: mesoventrite glabrous, mesepisternum and mesepimeron thinly covered with short pubescence. Middle disc of metaventrite brown, with sparse hairs. Last sternite of male with very deep “U” shape emarginate cavity reaching nearly its basal margin.

Legs moderately stout, hind tarsomere 1 nearly equal with last which is nearly as long as 2 and 3 together.

Male. Last abdominal sternite with very deep U-shape cavity (Fig. 46). Aedeagus: dorsal view: strongly asymmetrical but nearly parallel-sided, apex subacute, tapered; lateral view: somewhat sinuate on left side, gradually tapering to acute tip on right side. (Figs 48–49).

Female. Last abdominal sternite with triangle emargination at center of apex (Fig. 45). Spermatheca: base not bent, capsule wall thick, apex of capsule about 1/2 as long as capsule (Fig. 47).

Length: 5.4–5.5 mm (linear distance from labrum to elytral apex); width: 2.0–2.1 mm (width across base of elytra).

**Figures 44–49.**
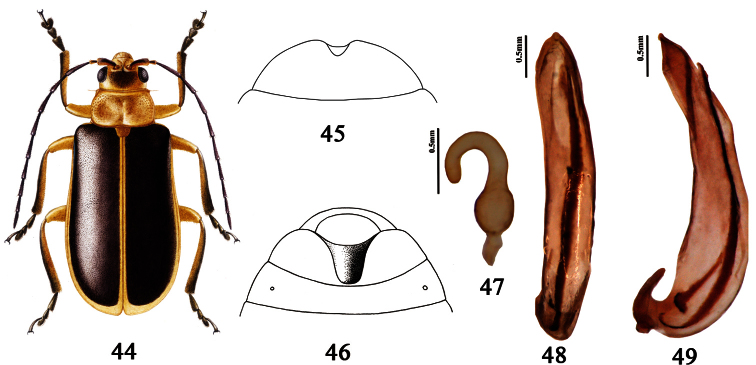
*Pyrrhalta quinana* sp. n. **44** Adult **45** The last abdominal sternite of female **46** The last sternite of male **47** spermatheca **48–49** aedeagus (**48** dorsal view **49** lateral view).

##### Etymology.

This species is named for its holotype locality, Guizhou province (shortened form as “Qian” in Chinese).

##### Distribution.

China (Guizhou, Sichuan).

## Discussion

*Pyrrhalta* is a species rich, worldwide distributed genus with complex classification and nomenclature. The relationships of *Pyrrhalta* and related genera (*Galerucella*, *Neogalerucella*, *Xanthogaleruca*, and *Tricholochmaea*) are still unclear. The updated catalogue of Galerucinae treated *Galerucella*, *Xanthogaleruca*, and *Tricholochmaea* were as valid genera and *Neogalerucella* as subgenus of *Galerucella* (Beenen 2010). To explore true relationship of these groups, a thorough revision of *Pyrrhalta* is necessary. Considering that subgenera currently are very poorly defined, we will separate the genus into several artificial groups and produce a series of revisionary works. In this study, we have reviewed *Pyrrhalta* species with black elytra. Based on the current morphological work with thirteen species of *Pyrrhalta* with black elytra two types of internal sac were found. One is with comb-shaped sclerites and another one lacking the sclerites. Comb-shaped sclerites presents in *Pyrrhalta sulcatipenni*s and *Pyrrhalta subaenea*. They are absent inthe rest of the studied species including *Pyrrhalta orientalis* which is placed in *Xanthogaleruca* in the updatedcatalogue of Galerucinae (Beenen 2010). Therefore, comb-shaped internal sac cannot be used to distinguish *Xanthogaleruca* and *Pyrrhalta*. The proper status of *Xanthogaleruca* could not resolve until a thorough revision of *Pyrrhalta* is done. We still need to find reliable characters to identify above related groups. It may be necessary to combine traditional morphological methods with molecular and biological methods to achieve this goal.

## Supplementary Material

XML Treatment for Pyrrhalta fossata

XML Treatment for Pyrrhalta huangshana

XML Treatment for Pyrrhalta martensi

XML Treatment for Pyrrhalta meghalayana

XML Treatment for Pyrrhalta metallica

XML Treatment for Pyrrhalta orientalis

XML Treatment for Pyrrhalta subaenea

XML Treatment for Pyrrhalta sulcatipennis

XML Treatment for Pyrrhalta tatesuji

XML Treatment for Pyrrhalta tianmuensis

XML Treatment for Pyrrhalta warchalowskii

XML Treatment for Pyrrhalta xizangana

XML Treatment for Pyrrhalta qianana
